# A Genome-Wide Association Study of Novel Genetic Variants Associated With Anthropometric Traits in Koreans

**DOI:** 10.3389/fgene.2021.669215

**Published:** 2021-05-13

**Authors:** Hye-Won Cho, Hyun-Seok Jin, Yong-Bin Eom

**Affiliations:** ^1^Department of Medical Sciences, Graduate School, Soonchunhyang University, Asan-si, South Korea; ^2^Department of Biomedical Laboratory Science, College of Life and Health Sciences, Hoseo University, Asan-si, South Korea; ^3^Department of Biomedical Laboratory Science, College of Medical Sciences, Soonchunhyang University, Asan-si, South Korea

**Keywords:** genome-wide association study, Korean, East Asian, *CUX2*, rs7133285, eQTL, anthropometric traits

## Abstract

Most previous genome-wide association studies (GWAS) have identified genetic variants associated with anthropometric traits. However, most of the evidence were reported in European populations. Anthropometric traits such as height and body fat distribution are significantly affected by gender and genetic factors. Here we performed GWAS involving 64,193 Koreans to identify the genetic factors associated with anthropometric phenotypes including height, weight, body mass index, waist circumference, hip circumference, and waist-to-hip ratio. We found nine novel single-nucleotide polymorphisms (SNPs) and 59 independent genetic signals in genomic regions that were reported previously. Of the 19 SNPs reported previously, eight genetic variants at *RP11-513I15.6* and one genetic variant at the *RP11-977G19.10* region and six Asian-specific genetic variants were newly found. We compared our findings with those of previous studies in other populations. Five overlapping genetic regions (*PAN2*, *ANKRD52*, *RNF41*, *HGMA1*, and *C6orf106*) had been reported previously but none of the SNPs were independently identified in the current study. Seven of the nine newly found novel loci associated with height in women revealed a statistically significant skeletal expression of quantitative trait loci. Our study provides additional insight into the genetic effects of anthropometric phenotypes in East Asians.

## Introduction

Human anthropometric traits, including height, body mass index (BMI), and fat distribution, differ substantially according to gender and genetic factors. Particularly, height, which has been associated with multiple diseases, is highly representative of the heritable phenotypic trait (Akiyama et al., 2019). Anthropometric traits related to obesity such as body size and composition are highly associated with metabolic syndrome (Wen et al., 2016). Furthermore, anthropometry is occasionally considered the traditional and basic tool of biological anthropology. However, it also plays an essential role in forensic science (Krishan, 2006; Thamizhselvi and Geetha, 2019).

A high degree of ethnic differences in adult anthropometric traits has been reported (Marigorta and Navarro, 2013). Many previous large-scale genome-wide association studies (GWAS) conducted in European populations revealed genetic variants correlated with various anthropometric traits. However, there is limited evidence supporting the variations associated with anthropometric traits in East Asian populations (Tachmazidou et al., 2017; Rask-Andersen et al., 2019). Therefore, given that various populations carry specific genetic variants, identification of genetic variants in large East Asian population samples is important in understanding the genetic determinants associated with anthropometric traits.

To date, more than 106 loci have been associated with anthropometric traits such as height and fat distribution (Tachmazidou et al., 2017). Tachmazidou et al. (2017) performed a GWAS of 12 anthropometric traits correlated with height and body mass in European populations and discovered six novel loci related to height and hip circumference (*CCDC36*, *HCG18*, *ZNF143*, *RP11-63E9.1*, *DDX51*, and *RP11-788M5.4*) and 28 independent genetic variants in previously reported genes. Randall et al. (2013) identified seven signals that were significant in women (located near *GRB14/COBLL1*, *LYPLAL1/SLC30A20*, *VEGFA*, *ADAMTS9*, *MAP3K1*, *HSD17B4*, and *PPARG*). More recently, Rask-Andersen et al. (2019) confirmed independent genetic signals related to the adiposity phenotype in the UK Biobank and identified clear differences between males and females, especially regarding fat distribution in the legs and trunk. Akiyama et al. (2019) performed a large-scale genetic association study and characterized 22 rare and 42 low-frequency height-associated single-nucleotide polymorphisms (SNPs) in a Japanese population.

Therefore, to explore the specific genetic signals of anthropometric traits in a Korean population, we performed a study using KoreanChip (KCHIP, Seoul, South Korea). The present study investigated the genetic factors related to the anthropometric phenotypes of 64,193 participants from two independent Korean cohorts. Here we performed GWAS for each gender to identify novel genetic variants reaching a genome-wide significance threshold (*p*-value < 1 × 10^–8^). We found not only nine novel SNPs that had not been reported previously, which were associated with height in females, but also 59 independent genetic signals in genomic regions that had been reported previously.

## Materials and Methods

### Study Design and Participants

The present study included two independent cohorts at the discovery and replication stages. The participants in the discovery stage (phase 1) were recruited from the Ansan/Ansung cohorts of the Korean Genome and Epidemiology Study (KoGES) between 2001 and 2002 (Kim et al., 2017), known as the Korea Association REsource (De Vries et al., 2019) project. The study involved 10,038 participants and included the genetic data of 5,493 participants (2,616 men and 2,877 women; age, 40–69 years). The participants in the replication stage (phase 2) were selected from the Health Examinee (HEXA) study cohort of the KoGES, which included a total of 173,357 participants recruited between 2004 and 2013 (Kim et al., 2017). This study included participants from urban (Seoul, Incheon, Daejeon, Daegu, Ulsan, Busan, and Gwangju) and rural (Gyeonggi, Sejong, Gangwon, Chungcheongbuk, Chungcheongnam, Gyeongsangbuk, Gyeongsangnam, Jeollabuk, Jeollanam, and Jeju) areas, and all participants were between 40 and 79 years of age. Among a total of 173,357 participants with baseline data in the HEXA study, only 58,700 participants were selected for the replication analysis. Height (cm), weight (kg), waist circumference (cm), and hip circumference (cm) were examined, and the BMI [weight (kg)/height (m^2^)] and waist-to-hip ratio (WHR = waist/hip) were computed. To reflect body fat distribution independent of overall adiposity, waist circumference, hip circumference, and the WHR were also analyzed and adjusted for BMI (waistBMIadj, hipBMIadj, and whrBMIadj). Since the anthropometric traits differed by gender in various aspects, males and females were analyzed separately (Randall et al., 2013). Participants with values greater than three standard deviations (SD) (depending upon cohort, sex, and trait) were also excluded from the study.

### Genotyping and Quality Control

Genotype data were provided by the Center for Genome Science, Korea National Institute of Health. DNA samples were separated and extracted from the peripheral blood of the participants. DNA genotyping of both the discovery and replication GWAS populations was performed using the Korea Biobank Array, which was designed by the Center for Genome Science, Korea National Institute of Health, South Korea, and referred to as the KoreanChip (KCHIP; Seoul, South Korea). The KCHIP array included a total of 833,535 single nucleotide variants for autosomal chromosomes (Han et al., 2021). The location of the genes was assigned according to the National Center for Biotechnology Information Human Genome Build 37 (hg19). The detailed KoreanChip analysis was reported previously (Moon et al., 2019; Jin et al., 2020). We excluded samples matching one of the following criteria: (i) genotyping accuracy less than 96–99% (Heid et al., 2010), (ii) excessive heterozygosity, and (iii) sex inconsistencies. SNPs were removed with (i) a missing call rate >5% (Heid et al., 2010), (ii) a minor allele frequency <1%, and (iii) a *p*-value in the Hardy–Weinberg equilibrium test <10^–4^. A total of 465 K variants were included after the quality control. After quality control and imputation, a total of 8,056,211 SNPs were used for this GWAS.

### Statistical Analysis

Most statistical analyses were performed using PLINK, version 1.90 beta^1^ (Purcell et al., 2007). Imputation of the genotype data was executed using IMPUTE v2 with data from the 1,000 genome phase 3 haplotypes serving as the reference panel (Chung et al., 2020; Oh et al., 2020). Only SNPs with an *r*^2^ value ≤ 95% with no linkage disequilibrium to each other were included in our study. GWAS were performed to identify SNPs associated with anthropometric traits *via* linear regression analysis with an additive model. Age and area were fitted as fixed covariates, and BMI was added to the adjustment as described above. The cutoff *p*-value suggesting the genome-wide significance level was *P* < 10^–5^ in the discovery stage (phase 1) and *P* < 10^––8^ in the replication and combined (discovery + replication) stages. GTEx Portal databases^2^ were used for expression quantitative trait loci (eQTL) analysis (GTEx Consortium, 2015), Haploview^3^ was used for Manhattan plots, and a regional plot was generated using LocusZoom^4^. Functional annotations such as protein motifs were analyzed using HaploReg^5^, and functional variants were identified by RegulomeDB^6^.

### Ethical Review

This study was approved by the Institutional Review Board of the Korea National Institute of Health (KBN-2021-003) and Soonchunhyang University (202012-BR-086-01). Written informed consent was obtained from all participants.

## Results

### Identification of Loci Related to Anthropometric Traits in the Discovery Stage

**FIGURE 1:**
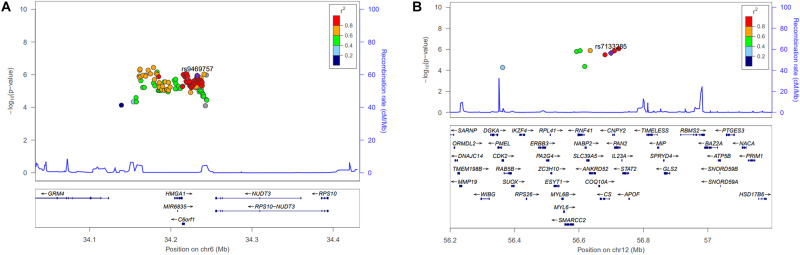
Regional association plot for the newly identified single-nucleotide polymorphisms (SNPs). Height-related signals in the discovery genome-wide association studies are plotted as –log_10_*P* values. The color of each SNP plot shows its linkage disequilibrium (using *r*^2^ values) with the novel SNP (purple diamond) within the association locus. The *y*-axis on the right shows the recombination rate from the HapMap database. The image above was constructed using the LocusZoom program (http://locuszoom.org/). **(A)** Regional plot of rs9469757 (*P* = 1.16 × 10^–6^) around the *RP11-513I15.6* region of chromosome 6 with height in women. **(B)** Regional plot of rs7133285 (*P* = 2.25 × 10^–6^) around the *RP11-977G19.10* region of chromosome 12.

The participants’ characteristics in the discovery and replication stages are listed in Table 1. We performed GWAS of 5,493 participants (2,616 men and 2,877 women) in the discovery stage and selected SNPs reaching the signal cutoffs for association at *P* < 10^–5^ and *P* < 10^–8^ in the discovery and replication stages, respectively. Manhattan plot showed genome-wide association between height and women in the discovery phase (Supplementary Figure 1). Nine novel SNPs, which were associated with height in women, located on autosomal chromosomes 6 and 12 at the genes *RP11-513I15.6* and *RP11-977G19.10* were identified, and eight genetic variants were found in *RP11-513I15.*6 (Table 2). In addition, 59 significant independent signals at previously reported regions and 19 signals identified in previous studies for different traits were found. Among the nine anthropometric traits, two male-related traits, including weight and WHR, and one female-related trait (height) reached a *p-*value < 10^–5^. The *HGMA1*, *C6orf106*, and *GRM4* genes reached the significance level for weight in men, and the *CUX2* gene reached significance for WHR in men (Supplementary Table 1). The genetic locations of these nine novel SNPs and their recombination rates in the discovery stage are plotted by their position in Figure 1. Moreover, the *LINC02456*, *GRM4*, *HMGA1*, *PAN2*, *SMIM29*, *C6orf106*, *ANKRD52*, *RNF41*, and *SLC39A5* loci reached the significance level for height in women. *HMGA1*, *C6orf106*, and *GRM4* were associated with anthropometric traits in both sexes. In particular, five genetic loci including rs1187115 (*P*_men_ = 1.73 × 10^–6^, *P*_women_ = 5.32 × 10^–7^), rs10807137 (*P*_men_ = 5.44 × 10^–6^, *P*_women_ = 6.88 × 10^–7^), rs370788671 (*P*_men_ = 6.85 × 10^–6^, *P*_women_ = 2.17 × 10^–6^), and rs9469745 (*P*_men_ = 9.74 × 10^–6^, *P*_women_ = 8.09 × 10^–6^) at *HMGA1* and rs6457765 (*P*_men_ = 5.53 × 10^–6^, *P*_women_ = 1.22 × 10^–6^) at *C6orf106* were identified as common anthropometric-related genetic variants in men and women.

**TABLE 1:** Characteristics of the study participants.

Characteristics	Men			Women		
	Discovery	Replication	Combination	Discovery	Replication	Combination
No.	2,616	20,293	22,909	2,877	38,407	41,284
Age (M years ± SD)	51.08 ± 8.33	55.18 ± 8.42	54.71 ± 8.51	51.98 ± 8.64	53.07 ± 7.70	53.00 ± 7.77
Height (M cm ± SD)	68.03 ± 9.46	168.69 ± 5.61	168.50 ± 5.65	153.98 ± 5.43	156.53 ± 5.16	156.36 ± 5.20
Weight (M kg ± SD)	167.00 ± 5.68	69.37 ± 8.72	69.22 ± 8.82	58.78 ± 7.94	57.62 ± 7.25	57.70 ± 7.31
BMI (M kg/m^2^ ± SD)	24.36 ± 2.84	24.37 ± 2.60	24.37 ± 2.63	24.78 ± 3.03	23.53 ± 2.78	23.62 ± 2.81
Hip circumference (M cm ± SD)	93.90 ± 5.59	95.71 ± 5.35	95.51 ± 7.28	93.38 ± 5.66	93.21 ± 5.36	93.22 ± 5.38
Waist circumference (M cm ± SD)	83.68 ± 7.45	85.62 ± 7.23	85.40 ± 7.28	81.40 ± 9.26	78.08 ± 7.87	78.31 ± 8.02
WHR	0.89 ± 0.06	0.89 ± 0.05	0.89 ± 0.05	0.87 ± 0.09	0.84 ± 0.06	0.84 ± 0.06

*BMI, body mass index; WHR, waist–hip ratio; *M*, mean value; SD, standard deviation.*

**TABLE 2:** Identified novel variants with genome-wide significant associations.

SNP	Nearest gene	Trait	Chromosome position	Minor allele	MAF	Discovery (*n* = 2,869)		Replication (*n* = 37,321)		Combination (*n* = 40,169)	
						β ± SE	*P* value	β ± SE	*P* value	β ± SE	*P* value
rs1307273	*RP11-513I15.6*	Height–women	6:34240886	G	0.205	0.82 ± 0.17	9.27 × 10^–7^	0.33 ± 0.04	5.19 × 10^–14^	0.37 ± 0.04	3.37 × 10^–17^
rs9469757	*RP11-513I15.6*	Height–women	6:34232864	C	0.170	0.87 ± 0.18	1.16 × 10^–6^	0.37 ± 0.05	1.41 × 10^–14^	0.40 ± 0.05	3.10 × 10^–17^
rs1759637	*RP11-513I15.6*	Height–women	6:34237130	C	0.193	0.82 ± 0.17	1.62 × 10^–6^	0.32 ± 0.05	4.49 × 10^–12^	0.35 ± 0.04	8.54 × 10^–15^
**rs7133285**	***RP11-977G19.10***	**Height–women**	**12:56699429**	**A**	**0.232**	**−0.75 ± 0.16**	**2.25 × 10^–6^**	**−0.67 ± 0.04**	**2.27 × 10^–54^**	**−0.67 ± 0.04**	**4.19 × 10^–58^**
rs9469761	*RP11-513I15.6*	Height–women	6:34236429	A	0.169	0.85 ± 0.18	2.53 × 10**^–^**^6^	0.37 ± 0.05	1.38 × 10**^–^**^14^	0.40 ± 0.05	3.25 × 10**^–^**^17^
rs1776890	*RP11-513I15.6*	Height–women	6:34237747	G	0.194	0.79 ± 0.17	3.62 × 10**^–^**^6^	0.32 ± 0.05	9.86 × 10**^–^**^13^	0.35 ± 0.04	1.51 × 10**^–^**^15^
rs13207853	*RP11-513I15.6*	Height–women	6:34238946	C	0.181	0.81 ± 0.18	4.64 × 10**^–^**^6^	0.33 ± 0.05	1.57 × 10**^–^**^12^	0.37 ± 0.05	1.59 × 10**^–^**^15^
rs2797961	*RP11-513I15.6*	Height–women	6:34237797	G	0.205	0.77 ± 0.17	4.67 × 10**^–^**^6^	0.32 ± 0.04	6.31 × 10**^–^**^13^	0.35 ± 0.04	9.07 × 10**^–^**^16^
rs200808496	*RP11-513I15.6*	Height–women	6:34237376	T	0.203	0.75 ± 0.17	7.40 × 10**^–^**^6^	0.31 ± 0.04	9.98 × 10**^–^**^12^	0.33 ± 0.04	5.34 × 10**^–^**^14^

*Age and residential area were included as covariants in all genetic models. Variants stated in the manuscript are indicated in bold. MAF, minor allele frequency; β, regression coefficient; SE, standard error; *N*, the number of participants included in each stage.*

### Genetic Variants Showing Association Signals in the Replication and Combination Stages

Single-nucleotide polymorphisms that reached a *p*-value < 10^–5^ in the discovery set were selected and re-evaluated in other stages. We performed a replication analysis of genetic variants found in the discovery stage, and finally, a total of 94 SNPs were analyzed in the combination stage. A total of 58,700 participants (20,293 men and 38,407 women) were included in the replication stage, and 64,193 participants (22,909 men and 41,284 women) were included in the combination stage. The genetic variants associated with waist circumference and BMI in the discovery stage did not meet the cutoff for signal association at *P* < 10^–5^ and *P* < 10^–8^ in both the discovery and replication stages. Moreover, a higher number of genetic signals were related to anthropometric traits in women than in men, and the statistical significance between SNPs and the traits was also higher in women. Among the nine novel SNPs related to height in women, rs7133285 in the *RP11-977G19.10* region had an even higher significance in the replication and combination stages compared with the discovery stage (*P*_rep_ = 2.27 × 10^–54^, *P*_*com*_ = 4.19 × 10^–58^) (Table 2). A similar trend was detected with the genetic loci rs146426492 (*P*_rep_ = 2.70 × 10^–53^, *P*_com_ = 4.07 × 10^–57^), rs72648137 (*P*_rep_ = 9.28 × 10^–48^, *P*_com_ = 7.81 × 10^–52^), rs76280383 (*P*_rep_ = 1.24 × 10^–29^, *P*_com_ = 2.00 × 10^–33^), and rs76459740 (*P*_rep_ = 2.23 × 10^–29^, *P*_com_ = 2.64 × 10^–33^), which were located on chromosome 12 and included in the 59 significant independent signals at previously reported regions and were definitely associated with height in women in the replication and combination stages.

### Functional Annotations of Associated Variants

**FIGURE 2:**
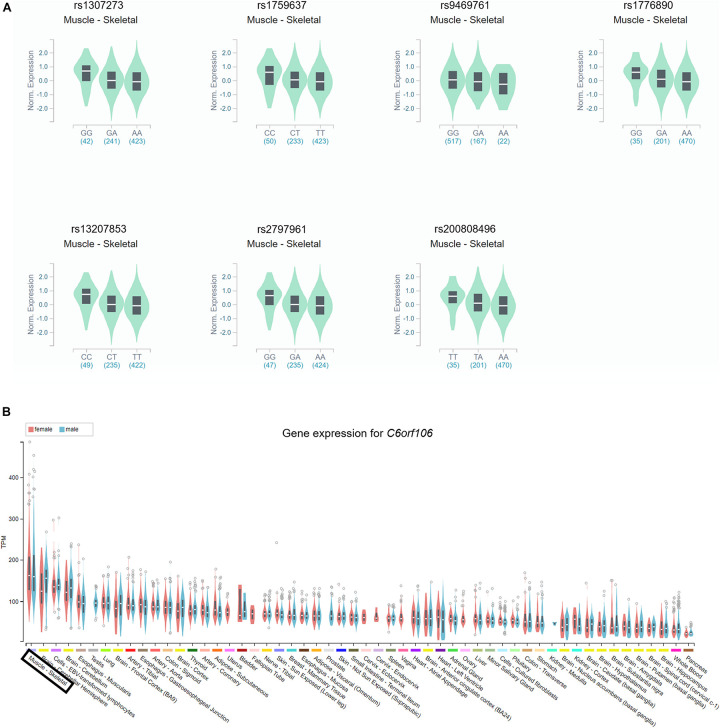
Identification of the gene expression of the *C6orf106* gene and seven novel SNPs in eQTL. The gene expression of each genotype in the skeletal muscle was presented using GTEx Portal and showed statistical significance. **(A)** Expression of each genotype of rs1307273, rs1759637, rs9469761, rs1776890, rs13207853, rs2797961, and rs200808496 in the RP11-513I15.6 region. All of the seven variants showed statistical significance reaching *P* < 10^–4^. **(B)** Gene expression of the *C6orf106* gene in the skeletal muscle.

Based on the GTEx databases, we analyzed the eQTL of the novel SNPs and new SNPs in previously reported regions. Among the nine newly found loci associated with height in women, statistically significant skeletal muscle eQTL were found for seven genetic variants (rs1307273, rs1759637, rs9469761, rs1776890, rs13207853, rs2797961, and rs200808496) (Figure 2A). In particular, rs1776890 and rs200808496, belonging to the skeleton eQTL, were associated with the expression of not only *NUDT3* but also *C6orf106* and *RPS10*. Additionally, we identified these seven height-related genetic variants as eQTL in subcutaneous adipose tissue that affects body fat distribution. Among the independent loci belonging to previously reported regions (*C6orf106*), rs6457765, rs147736074, and rs13201774 were eQTL expressed in the skeletal muscle (*P* = 6.80 × 10^–6^–9.50 × 10^–5^, eQTL effect size = −0.24 – −0.11) (Figure 2B, Supplementary Table 2, and Supplementary Figure 2).

### Geographic Distribution of Genomic Variants

**FIGURE 3:**
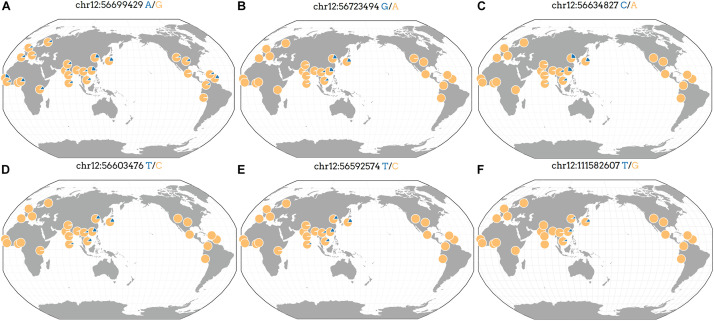
Geographic distribution of Asian-specific variants identified as independent signals in previously reported genes. The position of the genetic variant is described at the top, and the blue part indicates an allele frequency based on 1,000 genomes (hg19). **(A)** rs7133285, **(B)** rs146426492, **(C)** rs72648137, **(D)** rs76280383, **(E)** rs76459740, and **(F)** rs76892715.

We analyzed the frequency of rs7133285, rs146426492, rs72648137, rs76280383, and rs76459740 that showed a higher significance in the replication and combination stages than in the discovery stage. The GGV browser identifies the frequencies of the genetic variants in diverse populations based on 1,000 genomes (hg19) (Figure 3). The minor allele frequency of the rs7133285, which was distributed throughout the world, was higher in Africa and East Asia than in Europe or America (Figure 3A). Four variants, including rs146426492, rs72648137, rs76280383, and rs76459740, showed almost similar patterns, representing Asian genetic variants specific to the Asian population (Figures 3B–F). The minor allele frequencies in the cohort used in the present study are shown in Table 2 and Supplementary Table 1.

## Discussion

In the present study, we performed GWAS of anthropometric traits in a non-European population using KCHIP optimized for the Korean population (Moon et al., 2019) and presented the GWAS findings of anthropometric traits. Many studies related to anthropometric traits have been performed, and recent studies investigated Asian populations. However, few GWAS of anthropometric traits involving Korean population have been performed. Cho et al. (2009) performed a large-scale genetic association analysis of Koreans and found genetic factors related to eight quantitative traits including height, BMI, WHR, blood pressure, pulse rate, and bone density. The analysis was performed with Affymetrix5.0, which is a commercial SNP array designed for European or multiethnic populations (Cho et al., 2009; Kim et al., 2011). However, limitations in the capture of functional signals from next-generation sequencing were recently detected, including monomorphic variants in the Korean population. Therefore, we performed GWAS with KCHIP and identified nine novel genetic variants, 59 independent genetic signals in genomic regions that were reported previously, and 19 previously reported signals.

Overall, our results indicated similarities in the genetic signals associated with height in East Asians and Europeans. Among the 59 independent genetic signals in genomic regions that were reported previously (Supplementary Table 1), *C6orf106* (also known as *ILRUN*) (Weedon et al., 2008; Berndt et al., 2013; Tachmazidou et al., 2017; Kichaev et al., 2019), *GRM4* (Kichaev et al., 2019), *PAN2* (Allen et al., 2010), *SMIM29* (N’Diaye et al., 2011; Wojcik et al., 2019), and *ANKRD52* (Kichaev et al., 2019) were related to height in European populations. Besides this, *C6orf106*, which was related to weight in men in our study, also showed an association with lipid levels [high-density lipoprotein (HDL) cholesterol, low-density lipoprotein (LDL) cholesterol, and apolipoprotein A1 and B], metabolic syndrome, WHR-adjusted BMI, waist circumference, and BMI in diverse populations (De Vries et al., 2019; Richardson et al., 2020; Zhu et al., 2020). Except for the newly identified loci, 19 SNPs reached the cutoff for signal associations at *P* < 10^–5^ and *P* < 10^–8^ in the discovery and replication stages, respectively, that were previously reported for different or similar traits in other populations (Supplementary Table 3). In particular, rs671 belonging to *ALDH2* is a well-known variant related to metabolic syndrome and body mass in Asians (Wen et al., 2014; Zhu et al., 2017), and recently, Akiyama et al. (2019) reported that rs671 was associated with height in a Japanese population.

Analysis of a non-European population such as Korean for specific height-related signals including eight variants at the *RP11-513I15.6* region and one variant at the *RP11-977G19.10* region, which were not previously reported, may have an advantage. The comparison of a previous study of genetic variants influencing anthropometric traits in Koreans, using Affymetrix5.0, with the present study (Cho et al., 2009; Kim et al., 2010) showed that only the *HMGA1* region was associated with height in both studies. SNPs with higher statistical significance were found in the replication and combination stages compared with the discovery stage. Above all, the *PAN2* gene was found in an East Asian meta-analysis of GWAS, which interestingly revealed the highest statistical significance for adult height (He et al., 2015). Indeed, in a European GWA study, the *ANKRD52* gene, which was found to be associated with height and BMI (Kichaev et al., 2019; Zhu et al., 2020), showed significance in the replication and combined stages of the present study (Supplementary Table 1), and rs72648137 belonging to the *ANKRD52* gene in the present study was an Asian-specific genetic signal (Figure 3C). The genetic variant (rs76280383) belonging to the *RNF41* gene, which is known to be associated with congenital heart diseases in the Chinese Mongolian population (Zhang et al., 2016), was also an Asian-specific mutation (Figure 3D). Cho et al. (2009) stated that the genetic signals of anthropometric traits, especially BMI and height, overlapped with findings previously reported in a European population. Accordingly, the results of our study offer insights into the similarities and differences based on genetic factors associated with height and underscore the need for analysis of various populations to broaden our understanding of the genetic basis of anthropometric traits.

There were 16 independent signals related to weight or WHR of men at previously reported regions, which were located in four regions including *HGMA1*, *C6orf106*, *GRM4*, and *CUX2* (Supplementary Table 1). One study reported that the genetic variants in the *HGMA1* region were associated with an increased risk of type 2 diabetes (Bianco et al., 2015). Furthermore, *HGMA1* and *C6orf106* have been reported to show a genetic association with body fat ratios in Caucasians (Rask-Andersen et al., 2019), and both *GRM4* and *CUX2* were associated with the γ-GT-catalyzed reaction in excessive alcohol consumption (Chen et al., 2020). Additionally, the *CUX2* gene has been investigated in genome association studies related to serum uric acid levels, coronary artery disease, and hypertension (Cho et al., 2020; German et al., 2020; Matsunaga et al., 2020). However, to the best of our knowledge, the association with anthropometric trait (especially the WHR) GWAS signals has never been reported previously. Our GWAS demonstrated that the *CUX2* gene was associated with the WHR in Asian men (*P*_dis_ = 1.44 × 10^–6^, *P*_rep_ = 2.49 × 10^–15^, *P*_com_ = 4.63 × 10^–19^) and revealed the minor allele of rs76892715 in the *CUX2* gene in the East Asian population, but not in European, American, and African populations (Figure 3F).

In this study, there were significant genetic signals associated with weight and the WHR in males, but no new SNPs related to female fat distribution traits were found. However, previous studies, which analyzed the distribution of body fat and reported contradictory results, indicated that the genetic influence affecting fat distribution was more powerful in females than in males (Rask-Andersen et al., 2019). Other studies also revealed sexual dimorphism in the genetic effects related to fat distribution-related traits (Heid et al., 2010; Randall et al., 2013; Winkler et al., 2015). As shown in our results, rs76892715 in the *CUX2* region was associated with the WHR, a trait representing abdominal obesity in men (Supplementary Table 1). The *CUX2* gene is expressed higher in men than in women in general (Supplementary Figure 3), and males may be influenced more by the direct genetic association with *CUX2* than females. Further studies are required to elucidate these genetic signals between fat distribution and gender. Another limitation of our study was the lack of cohorts comprised of diverse populations and genotyping with the related assay chips, suggesting the need for further studies in the future.

In conclusion, we analyzed nine anthropometric traits and found nine novel genetic signals that had not been previously reported and 59 genetic independent variants in genomic regions that had been reported previously. Our study discovered novel loci in two regions including *RP11-513I15.6* and *RP11-977G19.10* associated with height in Korean women. Of the genetic loci previously associated with quantitative traits in non-Asian populations, 19 similar genetic variants that reached the cutoff for signal association were presented. Six Asian-specific genetic variants were also found, suggesting that both Asian and European populations show not only overlapping genetic signals but also characteristic anthropometric traits. Thus, anthropometric trait GWAS may enrich our perspective of anthropometric traits in East Asians, and optimization of ethnicity-specific genetic variants to distinguish nationality may contribute to the foundation of forensic anthropology.

## Data Availability Statement

The original contributions presented in the study are included in the article/Supplementary Material, further inquiries can be directed to the corresponding author.

## Ethics Statement

The studies involving human participants were reviewed and approved by the Institutional Review Board of the Korea National Institute of Health (KNIH and KBN-2021-003) and Soonchunhyang University (202012-BR-086-01). The patients/participants provided their written informed consent to participate in this study.

## Author Contributions

Y-BE and H-SJ participated in the design of the study, contributed to data reduction/analysis, and interpretation of the results. H-WC contributed to data analysis and interpretation of the results. All authors contributed to manuscript writing, reviewed and approved the final version of the manuscript, and agreed with the order of presentation of the authors.

## Conflict of Interest

The authors declare that the research was conducted in the absence of any commercial or financial relationships that could be construed as a potential conflict of interest.

## Footnotes

1 https://www.cog-genomics.org/plink2

2 https://gtexportal.org

3 https://www.broadinstitute.org/

4 http://locuszoom.org/

5 https://pubs.broadinstitute.org

6 https://regulomedb.org/

## Supplementary Material

Supplementary Figure 1 Manhattan plot showing a genome-wide association between height and women in the discovery stage. The red line suggests that the genome-wide significance level was of *p*-value < 10^–5^ in the discovery stage.

Supplementary Figure 2 Expression of each genotype of **(A)** rs6457765, **(B)** rs147736074, and **(C)** rs13201774 in the *C6orf106* region of the skeletal muscle.

Supplementary Figure 3 Gender-based gene expression of *CUX2*.

Supplementary Table 1 Identified independent signals at a previously reported gene with genome-wide significant associations.

Supplementary Table 2 Significant single tissue eQTL results for nine novel genetic variants and signals belonging to C6orf106 using the GTEx portal.

Supplementary Table 3 Replication of 19 previously discovered loci in our study populations.
