# Supplementary figures and images for: A Genome-Wide Association Study of Novel Genetic Variants Associated With Anthropometric Traits in Koreans
Source: Front Genet. 2021 May 13;12:669215. doi: 10.3389/fgene.2021.669215 (PMC8155599; doi:10.3389/fgene.2021.669215)

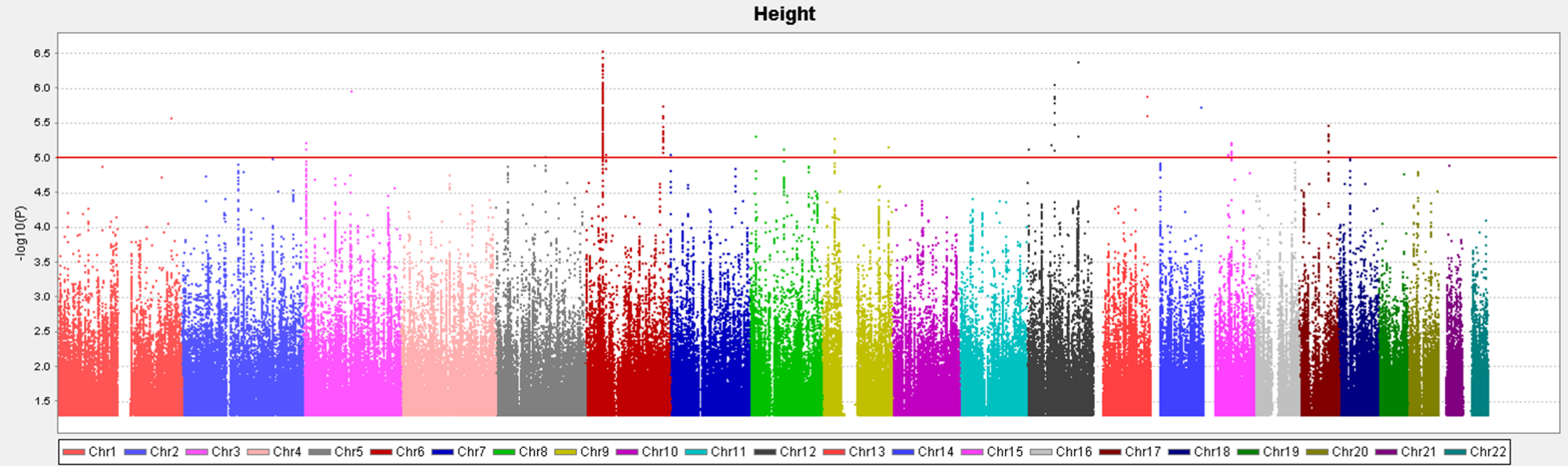

Supplement: Supplementary Figure 1 — Manhattan plot showing a genome-wide association between height and women in the discovery stage. The red line suggests that the genome-wide significance level was of p-value < 10–5 in the discovery stage. [file Image_1.TIF]

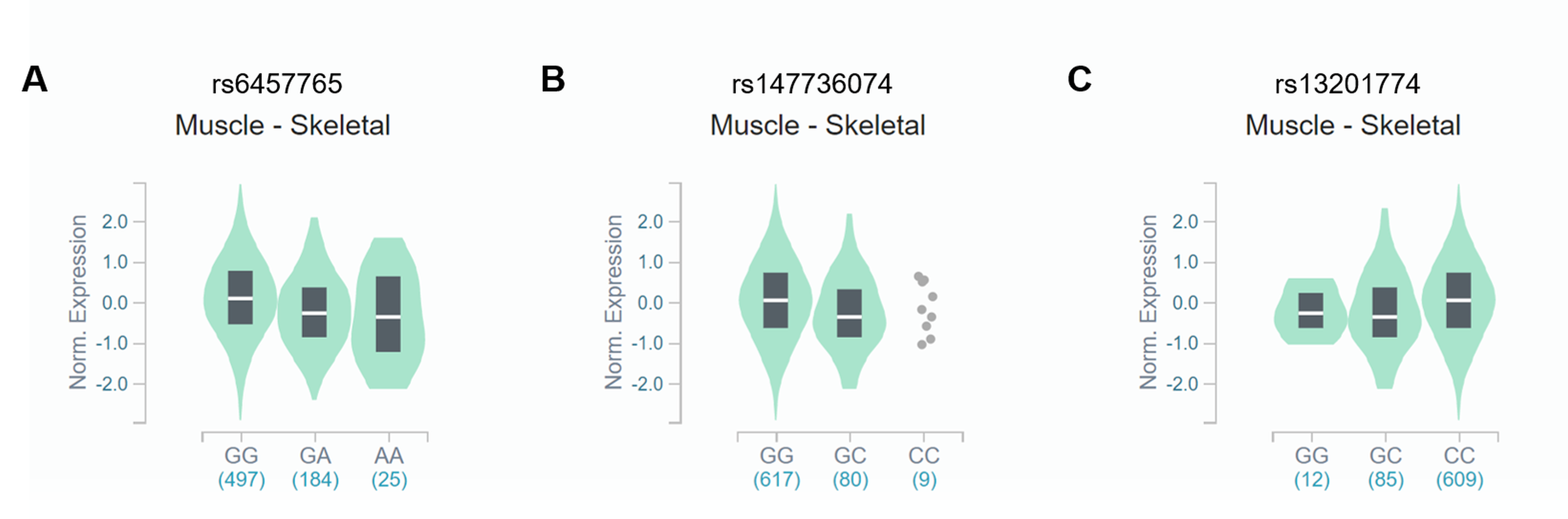

Supplement: Supplementary Figure 2 — Expression of each genotype of (A) rs6457765, (B) rs147736074, and (C) rs13201774 in the C6orf106 region of the skeletal muscle. [file Image_2.TIF]

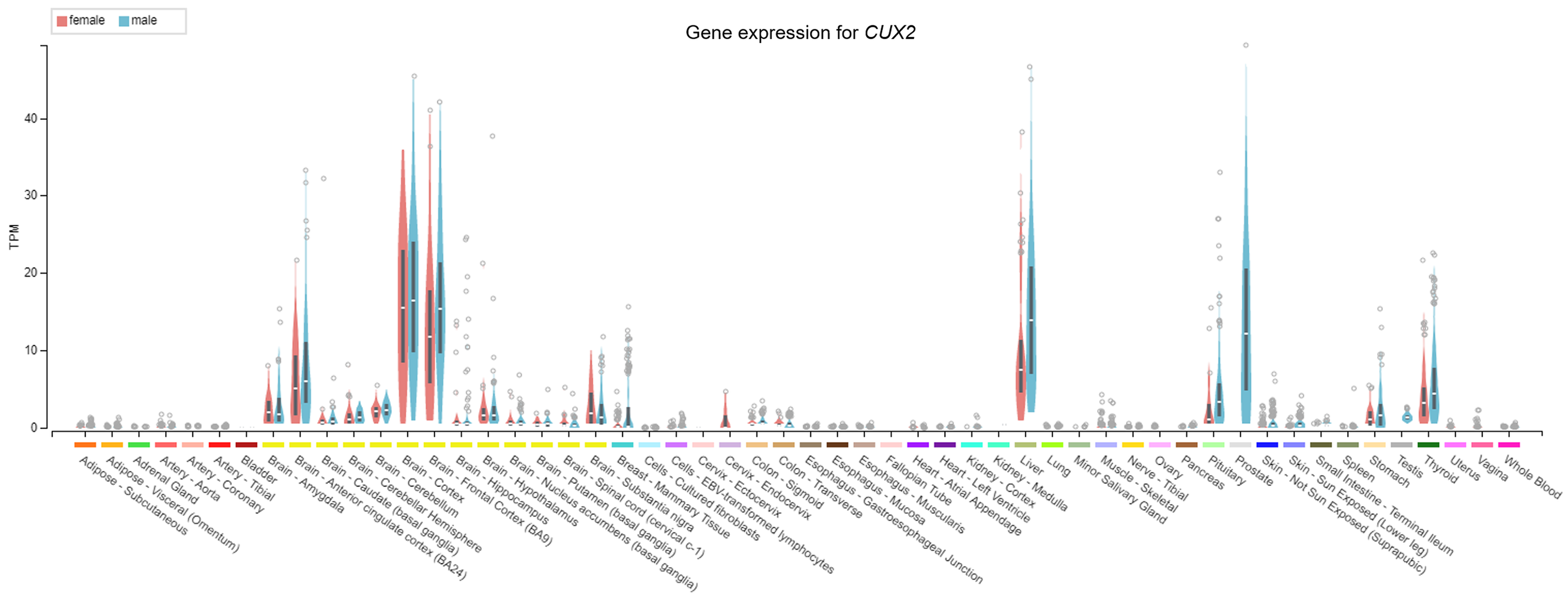

Supplement: Supplementary Figure 3 — Gender-based gene expression of CUX2. [file Image_3.TIF]
